# Factors influencing baseline electrocardiographic parameters in rats: A systematic review of experimental studies published from 1960 to the present

**DOI:** 10.14814/phy2.71131

**Published:** 2026-10-12

**Authors:** Viktória Novotná, Soňa Grešová, Pavol Švorc

**Affiliations:** ^1^ Department of Medical Physiology, Faculty of Medicine Pavol Jozef Šafárik University Košice Slovak Republic

**Keywords:** anesthesia, chronobiology, electrocardiography, rats, reference values, sex

## Abstract

Experimental studies in rats use baseline or control values as a reference against which changes after experimental interventions are assessed. However, baseline or control ECG parameters may already be influenced by several factors before the experiment begins, complicating interpretation of the results. Therefore, this review summarizes baseline or control ECG parameters (heart rate, PR (or PQ) interval, QRS complex, QT and QTc intervals, P‐wave duration and amplitude, R‐ and T‐wave amplitudes, and ST‐segment) from 285 in vivo rat studies published from 1960 to the present, identified through searches of Web of Science, Scopus, and PubMed. The aim of this review was not to assess the quality of the included studies, but to help researchers navigate the data and identify potential biological and methodological sources of variability or confounding affecting baseline or control ECG parameters. Our findings should be interpreted with caution due to limitations including variability and small datasets in some groups, unequal sample sizes, aggregation of study‐level mean values into a single mean, grouping of different anesthetic agents into a category, underrepresentation of females, incomplete reporting of anesthesia and measurement time, and inconsistent QTc correction methods. These limitations highlight the need for standardized experimental protocols and reporting practices.

## 1 INTRODUCTION

Electrocardiography (ECG) in rats is a widely used experimental method in basic cardiovascular research. Although the technique of ECG recording is relatively simple, the interpretation of ECG parameters can be challenging. Therefore, in in vivo studies involving rats, it is necessary to consider that baseline or control ECG values may be influenced by several factors, potentially affecting the validity and interpretation of the results even before the experimental intervention begins. However, no generally accepted criteria have been established for distinguishing physiologically relevant from insignificant changes in ECG parameters in rats, particularly with respect to baseline or control values. This concern was also highlighted by Konopelski and Ufnal (2016), who noted that differences in experimental conditions, particularly anesthesia, rat strain, and age, may contribute to variability in ECG parameters in rats (Konopelski & Ufnal, 2016). Another potentially important source of variability in baseline or control ECG parameters is acute emotional stress, which may induce autonomic changes with implications for cardiac electrophysiology (Mouchtouri et al., 2020).

Although general anesthesia is commonly required to minimize pain, stress, and movement in rats, it may also alter physiological responses relevant to ECG assessment. For example, anesthesia has been reported to disrupt the circadian rhythmicity of several physiological parameters, including heart rate (HR) (Orts‐Sebastian et al., 2019). Differences in the timing of ECG recordings may therefore represent another important source of inter‐study variability, particularly when the light/dark period or zeitgeber time is not consistently reported or controlled. In addition, different anesthetics may exert different effects on cardiomyocytes and the cardiac conduction system (Konopelski & Ufnal, 2016). The effects of anesthesia on cardiovascular parameters may depend not only on the anesthetic agent itself but also on the depth of anesthesia and body temperature. In male Sprague–Dawley (SD) rats, Redfors et al. (2014) demonstrated that different anesthetic regimens resulted in distinct cardiovascular responses, while changes in the concentration of isoflurane also affected cardiac function and blood pressure in a dose‐dependent manner. Furthermore, experimentally induced reductions in body temperature significantly decreased HR and cardiac output (Redfors et al., 2014).

Another potentially important source of variability is strain, body weight, and age. For example, differences in ECG parameters among rats of different strains and ages in hypertension models have been reported, suggesting that both genetic background and age‐related changes may contribute to variability in cardiac electrical activity (Conceição‐Vertamatti et al., 2020). Among normotensive controls, 6‐week‐old Wistar‐Kyoto (WKY) rats exhibited a shorter PR interval and a longer QRS duration than Wistar rats. By 15 weeks of age, the QT interval was also longer in WKY rats than in Wistar rats (Conceição‐Vertamatti et al., 2020). Across the age groups of 21 days and 2, 3, 7, and 18 months, Pezolato et al. (2017) observed a non‐uniform increase in QT interval duration, whereas the QTc interval corrected using the Bazett formula increased gradually from 21 days through 2, 3, 7, and 18 months of age in rats under pentobarbital anesthesia (Pezolato et al., 2017). An age‐related effect was also demonstrated under tribromoethanol anesthesia by Silva et al. (2002), who reported a twofold longer QTc interval in older rats than in young rats at relatively similar HR values (Silva et al., 2002). Prolongation of the QT and QTc intervals (Bazett formula) with increasing age was also observed in rats under pentobarbital anesthesia (Salari et al., 2024). Strutynska et al. (2025) further confirmed this tendency, reporting changes in QT and QTc intervals in rats under urethane anesthesia, with QTc corrected using the Sagie (Framingham) formula (Strutynska et al., 2025). Body weight may represent another source of variability in baseline ECG parameters. In Wistar rats, experimentally induced obesity was associated with an increased resting HR and shorter QRS and QT intervals, accompanied by changes in Q‐ and T‐wave amplitudes. Moreover, increasing body weight was positively correlated with resting HR and R‐wave amplitude, suggesting that body size and metabolic status may influence cardiac electrical activity (Mutiso et al., 2014).

Sex‐related differences should also be considered when interpreting baseline or control ECG findings in experimental studies. For example, telemetric ECG recordings in conscious Groningen rats revealed a significantly higher HR in females than in males (Carnevali et al., 2023). However, studies conducted under different anesthetic conditions have yielded inconsistent results, suggesting that observed sex‐related differences may also be influenced by the anesthetic regimen. For example, the PQ interval was longer in male SD rats than in females under barbital and light ether anesthesia (Wexler et al., 1973) and under ketamine/medetomidine anesthesia (Brisinda et al., 2006), whereas females exhibited longer PQ intervals under ketamine/xylazine (Palozi et al., 2020) and isoflurane anesthesia (Elkholey et al., 2021). Similar inconsistencies have been observed for other ECG parameters. Female Lewis rats showed a shorter P‐wave duration than males in telemetric recordings (Yaghouby et al., 2020), whereas under isoflurane anesthesia, P‐wave duration was additionally influenced by age (Elkholey et al., 2021). In contrast, QT and QTc intervals appear to be slightly longer in females than in males under pentobarbital and ether anesthesia (Wexler et al., 1973) and under ketamine/xylazine anesthesia (Palozi et al., 2020).

Interpretation of the QT interval in rats presents additional challenges because the T wave is not clearly separated from the QRS complex. Therefore, an accurate method for analyzing repolarization time in non‐anesthetized rats needs to be developed. However, based on available data, the relationship between the QT interval and HR in rodents (Menezes et al., 1988) remains controversial. Another serious problem with the QT interval is its dependence on HR, which complicates the search for the most suitable correction formula (Ahnve, 1985; Malik, 2002). To account for this and improve the reliability of QT interval measurements, three basic assumptions have been proposed (Davey, 2002). First, the formula must remove the HR dependence. Second, knowledge of the QT duration at a given HR must permit prediction of the QT duration at all other HRs, and third, ease of use in daily practice. Over time, several formulas have been developed and proposed for calculating the QTc interval (Botelho et al., 2019; Takahara et al., 2006). However, various correction formulas are often used in methodologies, which makes the current state of describing cardiac abnormalities quite unclear.

Based on the available evidence, the aim of this work was to report the mean numerical values of the extracted baseline or control ECG parameters (HR, PR (or PQ) interval, QRS complex, QT and QTc intervals, P‐wave duration and amplitude, R‐ and T‐wave amplitudes, and ST‐segment) from in vivo experimental studies involving rats published from 1960 to the present, and to identify factors that may contribute to their variability. Importantly, the aim of the present review was not to assess the methodological quality of the included studies. It is intended primarily for researchers working with laboratory rats to facilitate the interpretation of available data and to highlight potential biological and methodological sources of variability or confounding that may influence baseline or control ECG parameters in the initial stages of experimental studies.

## 2 MATERIALS AND METHODS

This study followed the Preferred Reporting Items for Systematic Review and Meta‐Analyses (PRISMA) statement (see Data S1 for PRISMA 2020 Checklist).

### 2.1 Eligibility criteria

The PICOS framework (Population, Intervention, Comparator, Outcomes, Study Design) guided the identification of inclusion criteria, with the following specifications: (1) Population: normotensive rats of both sexes (weighing ≥100 g, ≥5 weeks of age); (2) Intervention: ECG recordings obtained using bipolar limb lead II under general anesthesia or telemetry in awake rats; (3) Comparator: different recording conditions; (4) Outcomes: differences in control or baseline ECG parameters; (5) Study design: experimental studies.

Therefore, this work included studies that fulfilled the following inclusion criteria: experimental studies that clearly reported control or baseline numerical values of ECG parameters (HR [beats/min]) and duration of PR (PQ) interval, QRS complex, QT and QTc intervals (in ms), duration and amplitude of P wave (ms and mV), amplitudes of R and T waves (mV) and ST segment (depression or elevation in mV) measured in bipolar limb lead II with surface sensing using standard needle electrodes applied subcutaneously. Studies involving normotensive males and females, weighing ≥100 g, ≥5 weeks of age, with measurements performed with the animals under general anesthesia, or in awake rats using telemetry were included.

The exclusion criteria were as follows: publications in languages other than English; studies that included hypertensive rat strains or models of experimentally induced hypertension; and studies involving mixed populations without the possibility of separating normotensive control data. Studies that did not report extractable baseline or control values for ECG parameters, or reported ECG data only graphically or qualitatively, were also excluded. Studies in which measurements were not performed in the bipolar limb lead II, or in which the lead was not clearly defined, were also excluded. Finally, studies with insufficient descriptions of animal characteristics and those involving rats outside the specified inclusion criteria were excluded.

### 2.2 Information sources

A manual search of studies published from 1960 to the present was performed independently by two authors (P.Š., V.N.) in the Web of Science, Scopus, and PubMed databases using the keyword “ECG in rat”. The literature search was conducted in August 2025 and was limited to English‐language publications; therefore, only studies available up to the date of the search were considered for inclusion. Extended search strategies using Boolean operators, Medical Subject Heading (i.e., “MeSH”) terms, or combinations of more specific keywords (e.g., [“ECG” OR “electrocardiography”] AND [rat OR “Sprague‐Dawley” OR “Wistar‐Kyoto”]) were not used in this screening. This single‐keyword approach was intended solely to maximize the inclusion of relevant studies.

### 2.3 Search strategy, article screening, selection process and data collection

Two of the authors (V.N. and P.Š.) performed the independent initial manual extraction of the screened data using Microsoft Word (Microsoft Corporation, Redmond, WA, USA). The following data, including baseline or control ECG parameters, were extracted from each study: author(s); year of publication; breeding strain; sex; number of rats used; body weight; age; QTc interval correction method; and anesthesia used (in the case of conscious rats, these were telemetric records). In the next phase of data extraction from the retrieved publications, the authors (V.N., S.G., and P.Š.) independently verified the obtained data and their suitability for final inclusion in the study based on predefined inclusion and exclusion criteria. Ultimately, 341 studies reporting numerical ECG parameters were assessed for eligibility, and 285 were included in the final analysis. A flow‐diagram illustrating the study selection process is presented in Figure 1.

**FIGURE 1:**
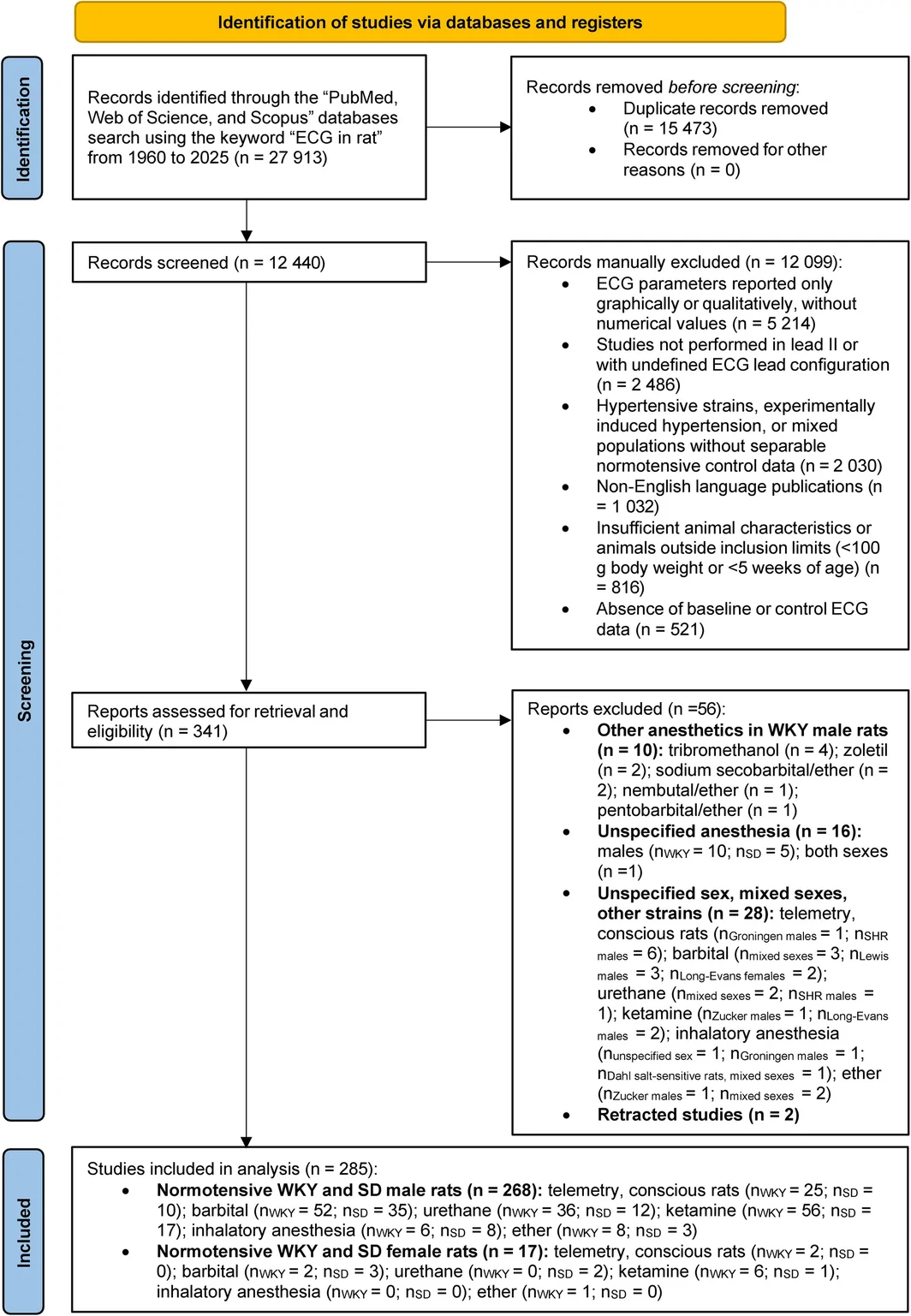
Flow diagram constructed in accordance with the PRISMA 2020 guidelines (Page et al., 2021). ECG, electrocardiography; SD, Sprague–Dawley; WKY, Wistar or Wistar/Kyoto.

### 2.4 Assessment of risk of bias

Because this review focused solely on the description of extracted mean ECG parameter values, the methodological quality of individual studies and risk of bias were not assessed using Cochrane's Risk of Bias Tool or any other validated scoring system.

### 2.5 Statistical analysis

The control or baseline values for individual ECG parameters were summarized, and the means with their corresponding standard deviations were calculated.

Due to the limited number of extracted mean ECG parameter values for some groups, anesthetics were divided into broader categories in the statistical analysis to ensure sufficient sample size and data comparability, as follows: (1) Conscious rats – non‐anesthetized conscious rats; (2) Barbital – nembutal, pentobarbital, thiopental, phenobarbitone, and thiopentone; (3) Ether – ether; (4) Ketamine – ketamine/xylazine, ketamine/xylazine/acepromazine, ketamine/midazolam, ketamine/diazepam, telazol/ketamine, medetomidine/ketamine, chlorpromazine/ketamine; (5) Urethane – urethane, urethane/α‐chloralose, chloral hydrate, α‐chloralose; and (6) Inhalation anesthesia – isoflurane, desflurane, sevoflurane, and enflurane.

For the same reason, hypertensive rats were excluded from the statistical analysis, and strain differences were assessed exclusively in normotensive rats, with the Wistar and Wistar/Kyoto (WKY) strains analyzed as a single group and compared with the SD strain due to the limited number of studies available for Wistar rats alone, which represents a significant limitation of the study. The following statistical tests were selected for data analysis:

1. Correlation analysis: The relationship between HR and QT interval length regardless of strain; the relationship between age and weight of females and males (in females, there was not a sufficient number of presented numerical values for a valid comparison of individual weight/age groups); and the relationship between weight and ECG parameters. In the case of correlation analysis and interpretation of Cohen's correlation coefficient, the following were considered: *r* = −0.3 to −0.5 as moderate negative dependence; *r* = −0.5 to −0.7 as large negative dependence; *r* = −0.7 to −0.9 as very large negative dependence; *r* = −0.9 to −1.0 = absolute negative dependence; *r* = 0.3–0.5 as moderate positive dependence; *r* = 0.5–0.7 as large positive dependence; *r* = 0.7–0.9 as very large positive dependence; *r* = 0.9–1.0 as absolute positive dependence.
2. Unpaired nonparametric Mann–Whitney test: The comparison of differences between WKY and SD rats in one specific ECG parameter and under one specific anesthetic group (e.g., conscious rats, barbital, ether, ketamine, urethane, and inhalation anesthesia). Therefore, it is not possible to conclude the effect of anesthesia from the results of this test, but only about differences between strains in the selected ECG parameter and the selected anesthetic.
3. Nonparametric Kruskal–Wallis test: A global assessment of the effect of individual anesthetics on the monitored ECG parameter within a specific strain. The resulting *p* value only indicates whether anesthesia has a statistically significant effect but does not provide information about differences between specific anesthetics (for this purpose, Dunn's post hoc test was subsequently performed).
4. Dunn's multiple comparison post hoc test: Identifying statistically significant differences between specific anesthetic categories within each normotensive WKY and SD strain.

For nonparametric tests, including Dunn's post hoc test, *p* < 0.05 was considered to be statistically significant. In the case of correlation analysis, data were analyzed by one author (P.Š.) using spreadsheet software (Excel, Microsoft Corporation, Redmond, WA, USA). The remaining statistical tests and figure generation were performed by another author (V.N.) using Prism version 8 (GraphPad Inc., San Diego, CA, USA).

For female rats, only descriptive mean values were extracted for WKY and SD rats without statistical comparison due to the limited number of studies involving female rats. For the same reason, differences between the sexes could not be statistically evaluated. As such, this work provides only descriptive/informative data on females. The above statistical comparisons did not account for QTc interval correction because the correction methods were inconsistent across the various studies or were not reported at all. Because most data sets exhibited non‐normal distributions according to the Shapiro–Wilk test, nonparametric statistical tests were considered most appropriate.

## 3 RESULTS OF THE LITERATURE REVIEW

### 3.1 QT–HR relationship and QT interval correction

As noted earlier, the relationship between the QT interval and HR in rodents remains controversial. Based on correlation analysis of the relationship between HR and the QT interval length, it was found that, with the most commonly used anesthetics in WKY and SD rats, except for urethane anesthesia (*r* = −0.35 [*n* = 16]), there is no statistically significant relationship (barbital, *r* = −0.1 [*n* = 38]; ketamine, *r* = −0.16 [*n* = 32]; inhalation anesthesia, *r* = −0.2 [*n* = 8]; and ether, *r* = 0.17 [*n* = 10]). Great caution is required when interpreting these results, as some correlation coefficients were calculated from a small number of extracted values and may not accurately reflect the underlying pathophysiological mechanisms (for example, Cohen's correlation coefficient for telemetric recordings in WKY rats, *r* = 0.6 [*n* = 4]). Another problem may be the fact that the dependence of QT interval duration on HR is described only in the light (i.e., inactive) period of the rat regime day. How the QT interval is affected by HR in the dark (i.e., active) period remains an open question.

To account for the dependence of the QT interval on HR, several correction formulas have been developed to calculate the QTc interval:

**Table:** 

Bazett: QTc = QT/√RR
Fridericia: QTc = QT (RR)^1/3^
Hodges: QTc = QT + 1.75 × (HR‐60)
Van der Water: QTc = QT + 0.087 (1‐RR)
Framingham, Sagie: QTc = QT + 0.154 (1‐RR)
Mitchell: QTc = QT/√(RR(100))
Matsunaga: QTc = log600 × QT/logRR

Various correction formulas are often used in methodologies, which makes the current state of describing cardiac abnormalities quite unclear. Often, authors describe changes in the QTc interval without specifying the actual numerical value or the correction formula used. This creates various discrepancies in the presentation of control values and the results themselves. Using individual correction formulas yields different values, which can be confusing and may not be informative, since standard reference values currently do not exist.

The actual use of correction formulas in the extracted studies for calculating the QTc interval in male WKY and SD rats, and females without specification of strain, is reported in Tables 1 and 2. In males, the Bazett formula was used in 44 cases (47.3% of the studies), Mitchell's formula in 3 (3.2% of the studies), Fridericia's formula in 4 (4.3% of the studies), the Framingham method in 2 (2.2% of the studies), and the Hodges formula in 1 (1.1% of the studies). As many as 39 studies (41.9% of the studies) did not describe the correction formula used, which represents a significant limitation of the interpretation of such in vivo studies. In females, the Bazett formula was used in three studies (42.9% of the studies), and no correction formula was mentioned in 4 (57.1% of the studies). In the available studies that corrected QTc using the Bazett formula (Table 1), a relatively marked variation in QTc was observed depending on the type of anesthesia. The mean values with standard deviation of the Bazett‐corrected QTc interval, including the number of included studies for each type of anesthesia and awake rats, are as follows:

**Table:** 

Barbital	171.33 ± 68.94 ms (*n* = 9)
Inhalation anesthesia	124.14 ± 36.99 ms (*n* = 5)
Urethane	109.88 ± 81.31 ms (*n* = 3)
Ketamine	139.29 ± 49.71 ms (*n* = 17)
Conscious rats	108.15 ± 46.78 ms (*n* = 5)

**TABLE 1:** Correction formulas applied in calculating the QTc interval in awake male rats and rats under various types of anesthesia, extracted from selected studies published between 1960 and the present.

	Authors
Bazett formula (*n* = 44; 47.3%)	
Conscious	Lujan et al. (2007); Corydon et al. (2020); Wood et al. (2010); Damasceno et al. (2012, 2013)
Barbital	Lessard et al. (1997); Pehlivan et al. (2020); Hughes (2020); Liu et al. (2014); Klimas et al. (2012); El Agaty and Seif (2015); Kumar et al. (2017); Lustyk et al. (2021, 2022)
Inhalation anesthesia	Ozturk and Altug (2007); Imoto et al. (2018, 2021); Letson et al. (2019); Wu et al. (2023)
Ketamine	Tylicki et al. (2008); Saeed et al. (2015); Basol and Erbas (2016); Salihi et al. (2017); Balea et al. (2018a, 2018b); Auzmendi et al. (2018); Boarescu et al. (2019); Durdagi et al. (2021); Rhana et al. (2022); Erbaş et al. (2015); Nwokocha et al. (2017); Bora et al. (2018); Kheiry et al. (2019); Sohrabi et al. (2020); Eltobshy et al. (2023); Sheibani, Nezamoleslami, Mousavi, et al. (2020)
Urethane	Umbarkar et al. (2015); Tomasova et al. (2016); Lin et al. (2021)
Tribromethanol	De Paula et al. (2017)
Ether	Manukhina et al. (2021)
Unspecified anesthesia	Al‐Asoom et al. (2014); Bavrina et al. (2015); Morand et al. (2018)
Mitchell formula (*n* = 3; 3.2%)	
Conscious	Maqoud et al. (2020)
Barbital	Shi et al. (2008)
Chloral hydrate	Gohma et al. (2006)
Fridericia formula (*n* = 4; 4.3%)	
Barbital	Maciel et al. (2010); Joukar (2012)
Ketamine	Akman et al. (2014)
Urethane	Sun et al. (2010)
Inhalation anesthesia	Patel and Brocks (2010)
Framingham formula (*n* = 2; 2.2%)	
Conscious	McCarroll et al. (2015)
Tribromethanol	Hadova et al. (2021, 2023)
Hodges formula (*n* = 1; 1.1%)	
Ketamine	Laska et al. (2024)
Unspecified formula (*n* = 39; 41.9%)	Silva et al. (2010); Medei et al. (2010); Lamb et al. (2012); Mamalyga (2013); Monnerat‐Cahli et al. (2014); Abood and Elshal (2015); Jafari et al. (2015); Temple et al. (2016); Binu et al. (2017); Asghari et al. (2017); Raji‐Amirhasani et al. (2018); Yadav et al. (2018); El‐Fawal et al. (2019); Aswar et al. (2019); Palozi et al. (2020); Ahmed et al. (2020); Haydari et al. (2020); Dietrichs et al. (2021); Rostamzadeh et al. (2022); Bocsan et al. (2021); Salama et al. (2021); Samadi et al. (2021); Barary et al. (2022); Rahimi Kakavandi et al. (2022); Hanna et al. (2022); Elkattawy et al. (2023); Beatch et al. (1991); Abbas and Sakr (2013); Xu et al. (2013); Naggar et al. (2014); Younis et al. (2017); Reno et al. (2017); Yang et al. (2019); Yang et al. (2020); Wang et al. (2020); Ling et al. (2021); Chowdhury et al. (2021); Hekmat et al. (2021); Gao et al. (2022)

*Note*: The table presents the number (*n*) and percentage of studies reporting each QT correction formula among the studies included in the QT correction formula analysis.

**TABLE 2:** Correction formulas applied in calculating the QTc interval in awake female rats and rats under various types of anesthesia, extracted from selected studies published from 1960 to the present.

	Authors
Bazett formula (*n* = 3; 42.9%)	
Conscious	Lujan et al. (2007)
Barbital	Volk et al. (2001)
Ketamine	Saklani et al. (2016)
Unspecified formula (*n* = 4; 57.1%)	Sosedova et al. (2019); Palozi et al. (2020); Saleh et al. (2020); Hamed et al. (2019)

*Note*: The table presents the number (*n*) and percentage of studies reporting each QT correction formula among the studies included in the QT correction formula analysis.

Several physiological and technical complexities and challenges prevent drawing clear conclusions regarding this issue based on the currently available data. Regarding the QTc interval, however, substantial evidence supports the concept that the widespread use of existing correction formulas can lead to significant variability; as such, further and more systematic investigation of this issue would be highly valuable (Mulla et al., 2022). Nevertheless, the QTc interval remains an important basic parameter for assessing electrical function of the heart due to its strong diagnostic utility.

### 3.2 Breeding strain

#### 3.2.1 Differences between male WKY and SD rats, regardless of anesthesia used

One factor that may affect ECG parameters is the breeding strain. The overall mean values of ECG parameters, regardless of anesthesia type and measurement method, in normotensive male WKY and SD rats indicate differences between the individual breeding strains. In males, the Mann–Whitney test revealed a statistically significant difference only in the duration of the QTc interval, which was longer in SD rats. The results approached statistical significance for the PR (PQ) interval, with higher mean values observed in SD rats. The mean values of the QRS complex and QT interval tended to be longer, although not significantly, in SD rats, while the mean HR was non‐significantly higher in WKY rats (Table 3, Figure 2). These findings should also be interpreted with caution because the data exhibit high variability that can be influenced by many of the factors discussed below. Therefore, the extracted ECG parameter values in Table 3 should be regarded as descriptive only. The mean values did not include data from male Long‐Evans (Mao et al., 1999; Medeiros et al., 2005), Lewis (Yaghouby et al., 2020; Yokokawa et al., 2008), Zucker (VanHoose et al., 2010), and Groningen rats (Rossi et al., 2014). However, methodological heterogeneity among the included studies, including differences in anesthesia, ECG recording conditions, level of stress, body weight, and QTc correction, as well as the combination of Wistar‐Kyoto and Wistar rats into one strain group limits the extent to which the observed differences can be attributed solely to strain.

**TABLE 3:** Mean values of ECG parameters in normotensive WKY and SD males regardless of the method of ECG recording and anesthesia used.

Parameter		HR (bpm)	PR (PQ) (ms)	QRS (ms)	QT (ms)	QTc (ms)
WKY	Mean ± standard deviation (*n*)	345.60 ± 69.66 (*n* = 125)	45.94 ± 8.48 (*n* = 98)	26.63 ± 14.05 (*n* = 114)	61.54 ± 21.52 (*n* = 106)	125.50 ± 62.67 (*n* = 73)
	95% CI	333.30–357.90	44.24–47.64	24.02–29.24	57.39–65.68	110.80–140.10
SD	Mean ± standard deviation (*n*)	339.0 ± 72.15 (*n* = 70)	48.65 ± 8.93 (*n* = 42)	27.32 ± 14.99 (*n* = 58)	63.28 ± 15.40 (*n* = 59)	158.0 ± 71.22 (*n* = 31)
	95% CI	321.80–356.20	45.87–51.43	23.38–31.27	59.26–67.29	131.9–184.1
Mann–Whitney *p* value		0.7370	0.0529	0.5582	0.2893	**0.0067**

*Note*: Electrocardiographic (ECG) data (HR, PR [PQ] interval, QRS complex, QT interval, and QTc interval irrespective of correction method) are presented as mean ± standard deviation, along with the number of extracted studies and the lower and upper 95% CI. Reported *p* values of the unpaired nonparametric Mann–Whitney test indicate differences between WKY and SD rats for each ECG parameter. Statistically significant differences (*p* < 0.05) are shown in bold.

Abbreviations: bpm, beats per minute; CI, confidence interval; HR, heart rate; ms, milliseconds; *n*, number of extracted mean values from selected studies; SD, Sprague–Dawley; WKY, Wistar or Wistar/Kyoto.

**FIGURE 2:**
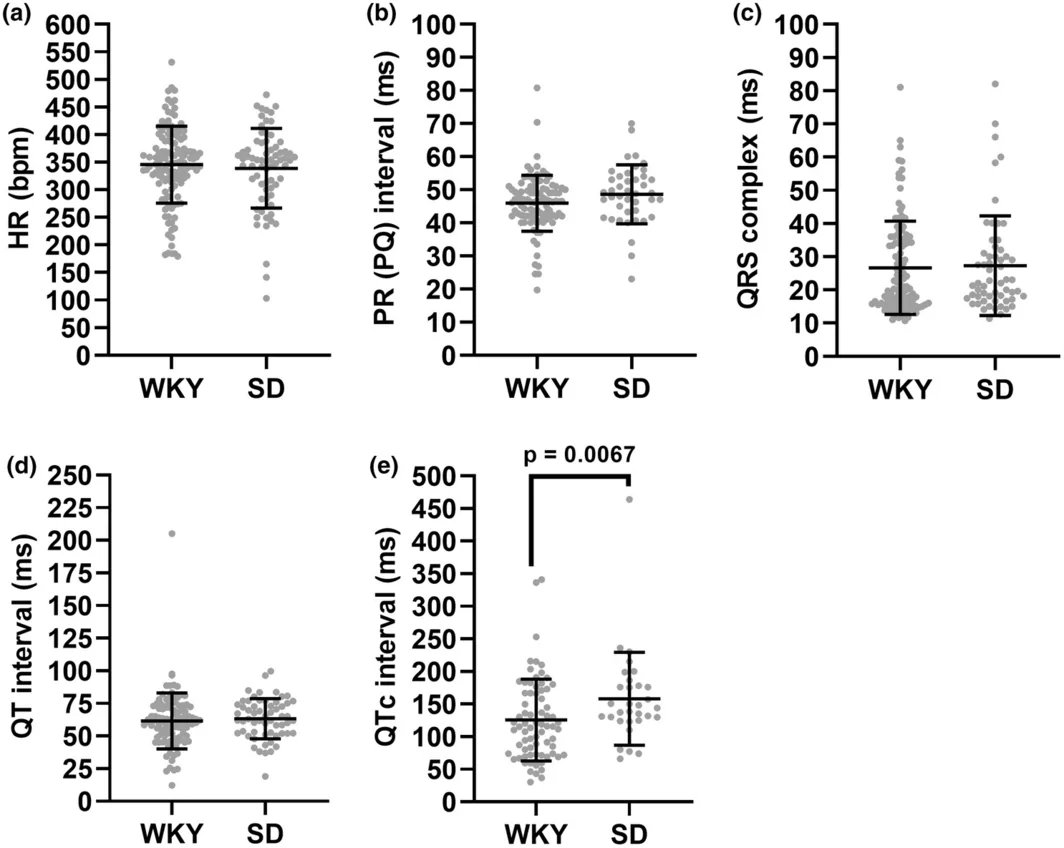
Electrocardiographic parameters in male WKY and SD rats, regardless of the anesthesia used. Differences in (a) HR (n_WKY_ = 125; n_SD_ = 70), (b) PR (PQ) interval (n_WKY_ = 98; n_SD_ = 42), (c) QRS interval (n_WKY_ = 114; n_SD_ = 58), (d) QT interval (n_WKY_ = 106; n_SD_ = 59), and (e) QTc interval (n_WKY_ = 73; n_SD_ = 31) between WKY and SD rats are shown as gray data points representing individual extracted mean values from available studies, with a black straight line indicating group means ± standard deviation. Statistically significant results according to the unpaired nonparametric Mann–Whitney test are indicated by bolded *p* values. bpm, beats per minute; HR, heart rate; ms, milliseconds; SD, Sprague–Dawley; WKY, Wistar or Wistar/Kyoto.

#### 3.2.2 Differences between female WKY and SD rats, regardless of the anesthesia used

In females, a valid statistical analysis could not be performed due to limited data extracted from the available studies; therefore, it was not possible to evaluate sex differences. However, Table 4 presents descriptive (i.e., informative) mean ECG values for female WKY and SD rats, regardless of the type of anesthesia or the method of QTc interval correction used. Mean HR and PR (PQ) interval values were higher in SD females, while QRS complex, QT and QTc intervals were longer in WKY females. These data are for informational purposes only and should be interpreted with caution due to the small number of extracted datasets and other limitations, similar to those discussed for males. A graphical representation of the differences between female WKY and SD rats is presented in Figure 3. Female Long‐Evans (Kopp et al., 1978; Kopp et al., 1980) and Lewis (Yaghouby et al., 2020) rats were not included in the mean values.

**TABLE 4:** Mean values of ECG parameters in normotensive female WKY and SD rats regardless of the method of ECG recording and anesthesia used.

Parameter		HR (bpm)	PR (PQ) (ms)	QRS (ms)	QT (ms)	QTc (ms)
WKY	Mean ± standard deviation (*n*)	317.50 ± 58.94 (*n* = 4)	47.50 ± 4.95 (*n* = 2)	24.45 ± 13.87 (*n* = 3)	68.33 ± 21.07 (*n* = 3)	164.90 ± 70.07 (*n* = 4)
	95% CI	223.70–411.30	3.028–91.97	−10.01–58.91	16.00–120.70	53.37–276.4
SD	Mean ± standard deviation (*n*)	351.70 ± 30.33 (*n* = 6)	52.40 ± 10.69 (*n* = 5)	17.81 ± 0.27 (*n* = 2)	66.72 ± 19.68 (*n* = 4)	60.00 ± 0.00 (*n* = 1)
	95% CI	319.8–383.5	39.13–65.67	15.40–20.22	35.40–98.04	—

*Note*: Electrocardiographic (ECG) data (HR, PR (PQ) interval, QRS complex, QT interval, and QTc interval irrespective of correction method) are presented as mean ± standard deviation, along with the number of extracted studies and the lower and upper 95% CI.

Abbreviations: bpm, beats per minute; CI, confidence interval; HR, heart rate; *n*, number of extracted mean values from selected studies; ms, milliseconds; SD, Sprague–Dawley; WKY, Wistar or Wistar/Kyoto.

**FIGURE 3:**
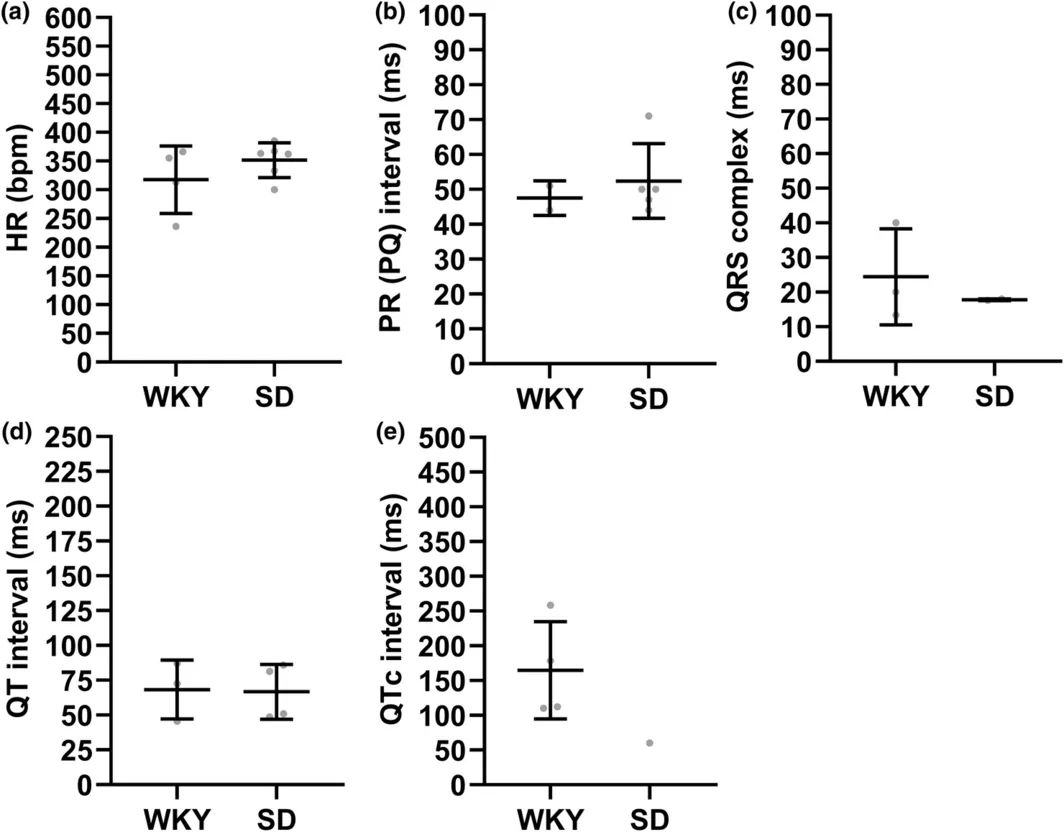
Electrocardiographic parameters in female WKY and SD rats, regardless of the anesthesia used, highlighting the underrepresentation of female cohorts in experimental studies. Differences in (a) HR (n_WKY_ = 4; n_SD_ = 6), (b) PR (PQ) interval (n_WKY_ = 2; n_SD_ = 5), (c) QRS complex (n_WKY_ = 3; n_SD_ = 2), (d) QT interval (n_WKY_ = 3; n_SD_ = 4), and (e) QTc interval (n_WKY_ = 4; n_SD_ = 1) between WKY and SD rats are shown as gray data points representing individually extracted mean values from available studies, with the black line indicating group means ± standard deviation. bpm, beats per minute; HR, heart rate; ms, milliseconds; SD, Sprague–Dawley; WKY, Wistar or Wistar/Kyoto.

### 3.3 Weight

When assessing the effects of weight on the monitored ECG parameters, we relied on the methodologies of individual studies that reported weight, which was sometimes quite misleading because the reported weight was not always directly related to the currently presented ECG parameter values. For weight ranges, the mean value within the stated range was determined. When assessing changes in ECG parameters depending on weight, we generated three weight categories for normotensive male rats: 100–200 g; 201–300 g; and ≥301 g. Unfortunately, the number of numerical values reported for females was insufficient for a representative comparison across individual weight groups. Age categories were generated based on the weight–age relationship derived from selected studies (Table 5).

**TABLE 5:** Average values of ECG parameters of male WKY and SD rats depending on weight, regardless of measurement method and type of anesthesia.

	100–200 g	201–300 g	≥301 g
WKY males			
HR (bpm)	334.16 ± 66.70 (*n* = 31)	343.22 ± 76.2 (*n* = 54)	365.67 ± 60.52 (*n* = 15)
PQ interval (ms)	44.37 ± 9.43 (*n* = 15)	44.75 ± 9.75 (*n* = 50)	48.28 ± 5.44 (*n* = 12)
QRS complex (ms)	29.73 ± 15.32 (*n* = 24)	27.24 ± 14.28 (*n* = 46)	21.86 ± 6.87 (*n* = 14)
QT interval (ms)	62.02 ± 15.99 (*n* = 22)	59.53 ± 16.00 (*n* = 51)	54.11 ± 25.68 (*n* = 10)
QTc interval (ms)	131.34 ± 42.59 (*n* = 11)	126.87 ± 61.9 (*n* = 35)	180.65 ± 26.39 (*n* = 6)
SD males			
HR (bpm)	341.08 ± 83.82 (*n* = 23)	347.60 ± 52.57 (*n* = 25)	301.63 ± 81.29 (*n* = 3)
PQ interval (ms)	47.48 ± 12.04 (*n* = 12)	47.64 ± 7.30 (*n* = 18)	56.76 ± 7.46 (*n* = 5)
QRS complex (ms)	27.01 ± 14.17 (*n* = 19)	22.46 ± 8.25 (*n* = 19)	25.77 ± 9.44 (*n* = 9)
QT interval (ms)	63.58 ± 18.39 (*n* = 20)	63.01 ± 15.23 (*n* = 25)	65.76 ± 11.45 (*n* = 8)
QTc interval (ms)	158.60 ± 36.95 (*n* = 11)	149.46 ± 48.55 (*n* = 13)	145.27 ± 32.85 (*n* = 3)

*Note*: Electrocardiographic (ECG) data (HR, PR (PQ) interval, QRS complex, QT interval, and QTc interval irrespective of correction method) are presented as mean ± standard deviation, along with the number of extracted studies (n).

Abbreviations: bpm, beats per minute; HR, heart rate; ms, milliseconds; *n*, number of extracted mean values from selected studies; SD, Sprague–Dawley; WKY, Wistar or Wistar/Kyoto.

Based on descriptive mean values extracted from the included studies (Table 5), HR tended to increase with increasing body weight in WKY rats. In SD rats, males weighing >301 g appeared to have lower mean HR values than those weighing 100–200 g and 201–300 g. In WKY rats, the PQ interval tended to lengthen, whereas QRS duration tended to shorten with increasing body weight. In SD rats, the PQ interval was longest in animals weighing >301 g, while QRS duration showed no clear pattern. The QT interval tended to shorten with increasing body weight in WKY rats, but not in SD rats. The QTc interval appeared to gradually lengthen with age in WKY rats, whereas it appeared to shorten in SD rats. The large dispersion of QTc values may indicate that different correction formulas were used across studies. It is important to note that some findings are based on very few studies and therefore represent only descriptive observations (Table 5).

### 3.4 Anesthesia

For determining reference values for HR, as well as other ECG parameters, the most logically suitable telemetric studies are those in which the rats are not under general anesthesia and the ECG can be recorded continuously during the day and in the awake state after the recovery period after the implantation of sensors (abdominal cavity or subcutaneously on the back). Telemetric studies reveal important information about fluctuations in myocardial electrophysiological parameters throughout the day. Currently, there are relatively few telemetric studies that analyze and report numerical values of ECG parameters in rats under in vivo conditions.

Mean values of ECG parameters from telemetry studies involving male WKY rats (Corydon et al., 2020; Damasceno et al., 2012; Damasceno et al., 2013; Kuberapandian & Doss, 2021; Kumar et al., 2017; Lamb et al., 2012; Lujan et al., 2007; Mamalyga, 2013; Maqoud et al., 2020; McCarroll et al., 2015; Monnerat‐Cahli et al., 2014; Nejm et al., 2020; Ngampongsa et al., 2011; Nijsen et al., 1998; Nijsen et al., 2000; Nijsen et al., 2001; Rey et al., 2012; Sanyal & Ono, 2002; Shimaoka et al., 2020; Temple et al., 2016; Thandapilly et al., 2013) as well as SD rats (Beatch et al., 1991; Chowdhury et al., 2021; Folkers et al., 1978; Ghelfi et al., 2010; Kuberapandian & Doss, 2021; Reno et al., 2017; Wood et al., 2010; Zhang et al., 2021) are presented in Table 6, together with the mean values of ECG parameters in WKY and SD rats measured in different anesthetic protocols.

**TABLE 6:** ECG parameters in male WKY and SD rats according to anesthesia used.

Conscious rats						
Parameter		HR (bpm)	PR (PQ) (ms)	QRS (ms)	QT (ms)*	QTc (ms)
WKY	Mean ± standard deviation (*n*)	349.20 ± 49.48 (*n* = 12)	48.06 ± 4.51 (*n* = 14)	29.10 ± 15.09 (*n* = 11)	61.38 ± 15.93 (*n* = 12)	121.80 ± 48.16 (*n* = 13)
	95% CI	317.70–380.60	45.45–50.67	18.97–39.24	51.26–71.50	92.69–150.90
SD	Mean ± standard deviation (*n*)	366.70 ± 20.83 (*n* = 5)	44.00 ± 4.24 (*n* = 2)	19.95 ± 3.58 (*n* = 6)	47.90 ± 0.00 (*n* = 1)	140.70 ± 58.84 (*n* = 4)
	95% CI	340.80–392.60	5.88–82.12	16.20–23.70	—	47.08–234.30
Mann–Whitney *p* value		0.2686	0.4000	0.4623	—	0.4912

Barbital anesthesia						
Parameter		HR (bpm)	PR (PQ) (ms)	QRS (ms)	QT (ms)	QTc (ms)
WKY	Mean ± standard deviation (*n*)	350.80 ± 61.30 (*n* = 38)	47.31 ± 7.13 (*n* = 35)	28.52 ± 16.79 (*n* = 37)	84.93 ± 50.36 (*n* = 36)	147.90 ± 72.74 (*n* = 20)
	95% CI	330.70–371.00	44.86–49.76	22.92–34.12	67.89–102.00	113.80–181.90
SD	Mean ± standard deviation (*n*)	363.10 ± 58.75 (*n* = 29)	47.97 ± 7.09 (*n* = 29)	31.93 ± 19.24 (*n* = 21)	61.06 ± 22.33 (*n* = 24)	177.90 ± 49.62 (*n* = 6)
	95% CI	340.80–385.50	44.55–51.38	23.17–40.69	51.63–70.49	125.80–230.00
Mann–Whitney *p* value		0.3867	0.9535	0.5025	**0.0230**	0.2425

Ether anesthesia						
Parameter		HR (bpm)	PR (PQ) (ms)*	QRS (ms)	QT (ms)	QTc (ms)*
WKY	Mean ± standard deviation (*n*)	367.50 ± 63.40 (*n* = 8)	45.62 ± 7.34 (*n* = 4)	31.50 ± 13.74 (*n* = 4)	80.42 ± 30.99 (*n* = 8)	68.80 ± 0.00 (*n* = 1)
	95% CI	314.50–420.50	33.94–57.30	9.63–53.37	54.51–106.30	
SD	Mean ± standard deviation (*n*)	360.00 ± 35.63 (*n* = 3)	50.97 ± 0.00 (*n* = 1)	30.55 ± 13.36 (*n* = 2)	69.43 ± 14.12 (*n* = 3)	186.3 ± 0.00 (*n* = 1)
	95% CI	271.50–448.50	—	−89.52–150.6	34.37–104.50	—
Mann–Whitney *p* value		0.8788	—	0.8000	0.8788	—

Ketamine anesthesia						
Parameter		HR (bpm)	PR (PQ) (ms)	QRS (ms)	QT (ms)	QTc (ms)
WKY	Mean ± standard deviation (*n*)	303.50 ± 79.46 (*n* = 27)	45.96 ± 11.08 (*n* = 24)	27.41 ± 16.73 (*n* = 30)	63.27 ± 18.62 (*n* = 33)	113.90 ± 46.18 (*n* = 26)
	95% CI	272.00–334.90	41.28–50.63	21.16–33.65	56.66–69.87	95.29–132.60
SD	Mean ± standard deviation (*n*)	256.30 ± 75.98 (*n* = 14)	51.23 ± 13.84 (*n* = 10)	24.90 ± 7.82 (*n* = 12)	70.07 ± 10.60 (*n* = 15)	187.9 ± 105.5 (*n* = 9)
	95% CI	212.40–300.20	41.33–61.13	19.93–29.87	64.20–75.94	106.8–269.0
Mann–Whitney *p* value		0.0966	0.1246	0.7889	0.1822	**0.0045**

Urethane anesthesia						
Parameter		HR (bpm)	PR (PQ) (ms)	QRS (ms)	QT (ms)	QTc (ms)
WKY	Mean ± standard deviation (*n*)	344.10 ± 63.31 (*n* = 25)	42.82 ± 9.30 (*n* = 14)	22.08 ± 14.25 (*n* = 23)	49.38 ± 15.92 (*n* = 17)	104.00 ± 58.00 (*n* = 6)
	95% CI	318.00–370.30	37.45–48.19	15.91–28.24	41.20–57.57	43.10–164.80
SD	Mean ± standard deviation (*n*)	359.70 ± 60.25 (*n* = 10)	46.39 ± 15.25 (*n* = 3)	28.44 ± 18.96 (*n* = 8)	66.11 ± 9.94 (*n* = 8)	136.00 ± 42.97 (*n* = 6)
	95% CI	316.60–402.80	8.50–84.28	12.59–44.29	57.81–74.42	90.86–181.10
Mann–Whitney *p* value		0.6795	0.7529	0.3330	**0.0085**	0.1797

Inhalation anesthesia						
Parameter		HR (bpm)	PR (PQ) (ms)	QRS (ms)	QT (ms)	QTc (ms)
WKY	Mean ± standard deviation (*n*)	393.80 ± 60.81 (*n* = 6)	45.48 ± 4.76 (*n* = 6)	23.60 ± 7.79 (*n* = 6)	62.35 ± 10.88 (*n* = 6)	139.4 ± 65.42 (*n* = 4)
	95% CI	330.00–457.60	40.49–50.48	15.43–31.77	50.93–73.77	35.25–243.4
SD	Mean ± standard deviation (*n*)	336.60 ± 35.16 (*n* = 5)	48.95 ± 1.97 (*n* = 4)	24.45 ± 12.82 (*n* = 5)	62.19 ± 5.61 (*n* = 5)	99.86 ± 26.03 (*n* = 4)
	95% CI	292.90–380.20	45.82–52.08	8.53–40.37	42.81–81.57	58.45–141.30
Mann–Whitney *p* value		0.0887	0.1143	0.9307	>0.9999	0.3429

*Note*: Electrocardiographic (ECG) data (HR, PR (PQ) interval, QRS complex, QT interval, and QTc interval irrespective of correction method) are presented as mean ± standard deviation, along with the number of extracted studies and the lower and upper 95% CI. Differences between male WKY and SD rats were evaluated under different anesthetic agents which were categorized into broader groups as follows: conscious rats, barbital, ether, ketamine, urethane, and inhalation anesthesia. Reported *p* values for the unpaired nonparametric Mann–Whitney test indicate differences between WKY and SD rats for each ECG parameter under different anesthetic conditions. ECG parameters for which statistical comparison could not be performed due to the limited number of extracted mean values (*n* = 1) should be considered descriptive or exploratory only and are marked with an asterisk (*). Statistically significant differences (*p* < 0.05) are reported in bold.

Abbreviations: bpm, beats per minute; CI, confidence interval; HR, heart rate; ms, milliseconds; *n*, number of extracted mean values from selected studies; SD, Sprague–Dawley; WKY, Wistar or Wistar/Kyoto.

The mean HR from telemetry studies in male WKY rats was 349.20 ± 49.48 beats/min, regardless of weight or time of day at which the experiments were performed. In male SD rats, the mean HR value was 366.70 ± 20.83 beats/min. The variability in HR was greater in male WKY rats than in male SD rats, as indicated by the larger standard deviation; however, HR in both strains fluctuates within the reported resting range of 250 to 400 beats/min.^1^ Differences in control values between WKY and SD rats should be considered with great caution because there are very few reported numerical values, especially in SD control groups.

The relatively large dispersion of control HR values can also be attributed to the effect(s) of anesthesia because some studies start from bradycardic values at the beginning of the experiment, in which HR was <200 beats/min. (Ahmed et al., 2021; Chakraborty et al., 2017; Farrag et al., 2023; Khorrami et al., 2014; Martín Barrasa et al., 2008; Omar et al., 2021; Silva et al., 2010) mainly under ketamine anesthesia. Control HR values >400 beats/min have been reported in many studies using barbital (Chen et al., 2007; Chen et al., 2018; Dietrichs et al., 2021; Hekmat et al., 2021; Howard et al., 1990; Huang et al., 2018; Joukar et al., 2013; Plotnikova et al., 2018; Pugsley et al., 2019; Schoors et al., 1991; Wang et al., 2018), inhalation anesthesia (Imoto et al., 2018; Ozturk & Altug, 2007), urethane (Aydin et al., 2016; Dai et al., 2018; Hu et al., 2015; Tiwari et al., 2009; Younis & Mohamed, 2022), ether (Manukhina et al., 2021; Viswanatha Swamy et al., 2013), ketamine (Kalkan et al., 2018; Saeed et al., 2015; Sedmera et al., 2016), and zoletil anesthesia (Ivonin et al., 2022a, 2022b). In one case, the tachycardic baseline value was at the beginning of the experiment in awake animals (Doss & Kuberapandian, 2021). This would mean that, at the very beginning of the experiment, with bradycardic values, there may be a potential risk for destabilization of repolarization and, with tachycardic values, if an abnormal substrate is present, the basis for the development of arrhythmia may be formed.

Unfortunately, there are also studies that did not report the type of anesthesia (Akhondali et al., 2015; Akila et al., 2017; Al‐Asoom et al., 2014; Aykan et al., 2024; Gaafar et al., 2017; Imbaby et al., 2014; Karimkhani et al., 2021; Naggar et al., 2014; Safaeian et al., 2023; Taskin et al., 2016; Yang et al., 2019; Yang et al., 2020; Zhang et al., 2022), although they describe changes in anesthetized animals. The question is, what are the reference values for ECG parameters in rats under non‐stressful circumstances under different types of anesthesia? Based on the values reported in Table 6, it is clear that ECG parameters vary with the type of general anesthesia, which can complicate the final assessment of changes in the monitored parameter after various interventions.

We considered the most common anesthetics used to induce general anesthesia, including barbital in WKY rats (Abdolghaffari et al., 2015; Abood & Elshal, 2015; Ahmed et al., 2020; Aimoto et al., 2022; Ashour et al., 2011; Bacharova et al., 2008; Binayi et al., 2016; Comerma‐Steffensen et al., 2017; Dietrichs et al., 2021; Dong et al., 1993; El Agaty & Seif, 2015; Elgebaly et al., 2023; Elkattawy et al., 2023; El‐Marasy et al., 2020; Fatahi et al., 2022; Fields, 1970; Haydari et al., 2020; Howard et al., 1990; Howarth et al., 2011; Hughes, 2020; Joukar, 2012; Joukar et al., 2013; Klimas et al., 2012; Korkmaz‐Icöz et al., 2021; Kráľová et al., 2008; Kumar et al., 2017; Lee et al., 1999; Lessard et al., 1997; Lustyk et al., 2021; Lustyk et al., 2022; Maatz et al., 2009; Machida et al., 1990; Maciel et al., 2010; Mohammed et al., 2020; Mousa et al., 2018; Pehlivan et al., 2020; Plotnikova et al., 2018; Rahimi Balaei et al., 2010; Raji‐Amirhasani et al., 2018; Schoors et al., 1991; Shi et al., 2008; Shumilova et al., 2010; Strinic et al., 2017; Suzuki et al., 1991; Van Buren et al., 1998; Vidal et al., 2010; Yokokawa et al., 2008) and SD rats (Ali et al., 2023; Barrett et al., 2000; Baur & Pierach, 1979; Çetin, 2019; Chen et al., 2007; Cheng et al., 2019; Elsherbiny et al., 2016; Gandhi et al., 2013; Gao et al., 2022; Ghelfi et al., 2008; Hekmat et al., 2021; Huang et al., 2018; Khalifa et al., 2022; Liu et al., 2014; Murakami et al., 2014; Ping et al., 2021; Ping et al., 2022; Pugsley et al., 1993; Pugsley et al., 2015; Pugsley et al., 2019; Rahmanifard et al., 2021; Salem et al., 2023; Su et al., 2021; Waite et al., 1990; Wang et al., 2021; Wang et al., 2023; Wexler et al., 1973; Wexler & Greenberg, 1974; Yang et al., 2020; Zhang et al., 2019; Zhang et al., 2022). Another commonly used anesthetic is urethane in WKY rats (Abd El‐Hakam et al., 2022; Al‐Ali et al., 2024; Amin et al., 2021; Aswar et al., 2019; Aydin et al., 2016; Beladiya et al., 2019; Bestetti et al., 1987; Buschmann et al., 1980; El‐Fawal et al., 2019; El‐Kharashi et al., 2019; El‐Shaer et al., 2021; Gohma et al., 2006; Hocaoglu et al., 2012; Liu et al., 2022; Parasuraman et al., 2011; Patil et al., 2018; Sabharwal et al., 2004; Salama et al., 2021; Sharma et al., 2018; Sonawane et al., 2018; Sultan et al., 2020; Sumbul & Aygun, 2022; Tiwari et al., 2009; Tomasova et al., 2016; Umbarkar et al., 2015; Warpe et al., 2015; Younis, 2022; Younis et al., 2020; Younis & Mohamed, 2022), and in SD rats (Ammar el et al., 2013; Badole et al., 2015; Dai et al., 2018; El‐Said et al., 2019; Emeka, 2017; Hu et al., 2015; Jain et al., 2018; Lin et al., 2021; Shaikh & Bhatt, 2022; Sun et al., 2010; Wang et al., 2020; Xu et al., 2013).

For ketamine anesthesia, we summarized and averaged data for anesthesia with ketamine alone as well as combinations such as ketamine/xylazine, ketamine/xylazine/acepromazine, ketamine/midazolam, ketamine/diazepam, ketamine/medetomidine, ketamine/chlorpromazine, and medetomidine/midazolam/butorphanol in WKY rats (Agarwal et al., 2021; Ahmadi et al., 2018; Akman et al., 2014; Arini et al., 2018; Asghari et al., 2017; Auzmendi et al., 2018; Aygun & Gul, 2019; Balea et al., (2018a, 2018b); Barary et al., 2022; Basol et al., 2019; Basol & Erbas, 2016; Bilginoğlu et al., 2014; Binu et al., 2017; Boarescu et al., 2019; Bocsan et al., 2021; Chakraborty et al., 2017; Durdagi et al., 2021; Firoz et al., 2011; Hanna et al., 2022; Jafari et al., 2015; Kalkan et al., 2018; Khorrami et al., 2014; Lima et al., 2022; Maleki et al., 2019; Medei et al., 2010; Mutiso et al., 2014; Omar et al., 2021; Palozi et al., 2020; Puri et al., 2005; Rahimi et al., 2018; Rahimi Kakavandi et al., 2022; Rhana et al., 2022; Rostamzadeh et al., 2022; Saeed et al., 2015; Salihi et al., 2017; Samadi et al., 2021; Sedmera et al., 2016; Sedyakina et al., 2024; Selcuk et al., 2015; Sheibani, Nezamoleslami, Faghir‐Ghanesefat, et al., 2020; Sheibani, Nezamoleslami, Mousavi, et al., 2020; Tylicki et al., 2008; Yadav et al., 2018) and in SD rats (Bello et al., 2023; Bora et al., 2018; Cagiltay et al., 2021; Eltobshy et al., 2023; Erbaş et al., 2015; Farrag et al., 2023; Jalal et al., 2023; Kheiry et al., 2019; Laska et al., 2024; Martín Barrasa et al., 2008; Naggar et al., 2014; Nwokocha et al., 2017; Sarihan et al., 2015; Shahsavand et al., 2020; Sohrabi et al., 2020).

Control values have also been reported for inhalation anesthesia in WKY rats (Botelho et al., 2019; Imoto et al., 2018; Imoto et al., 2021; Lebsir et al., 2020; Ozturk & Altug, 2007) and in SD rats (Guéguen et al., 2008; Jiang et al., 2014; Letson et al., 2019; Ling et al., 2021; Murakami et al., 2014; Patel & Brocks, 2010; Wu et al., 2023). Due to the small number of numerical values for SD rats, it was relatively difficult to evaluate changes in the monitored ECG parameters.

Although ether is no longer used to induce general anesthesia, few studies have used light ether anesthesia to record ECG in WKY rats (El‐Sayed et al., 2021; El‐Wakf et al., 2024; Farag et al., 2021; Fields, 1970; Hijazi et al., 2019; Keung & Aronson, 1981; Manukhina et al., 2021; Upaganlawar & Balaraman, 2010; Viswanatha Swamy et al., 2013) and SD rats (Abbas & Sakr, 2013; Normann et al., 1961; Patel et al., 2010). In some experiments, tribromoethanol (De Paula et al., 2017; Hadova et al., 2021; Hadova et al., 2023) as well as zoletil anesthesia (Ivonin et al., 2022a, 2022b) were used.

Table 6 also presents the results of Mann–Whitney tests comparing ECG parameters between WKY and SD rats under different anesthetics, including the awake state. Figure 4 illustrates the differences between WKY and SD rats in individual ECG parameters under different anesthetics, including awake animals (assessed using the Mann–Whitney test), as well as the differences between anesthetics within a particular strain, both in WKY and in SD, assessed using Dunn's post hoc test following the nonparametric Kruskal–Wallis test.

**FIGURE 4:**
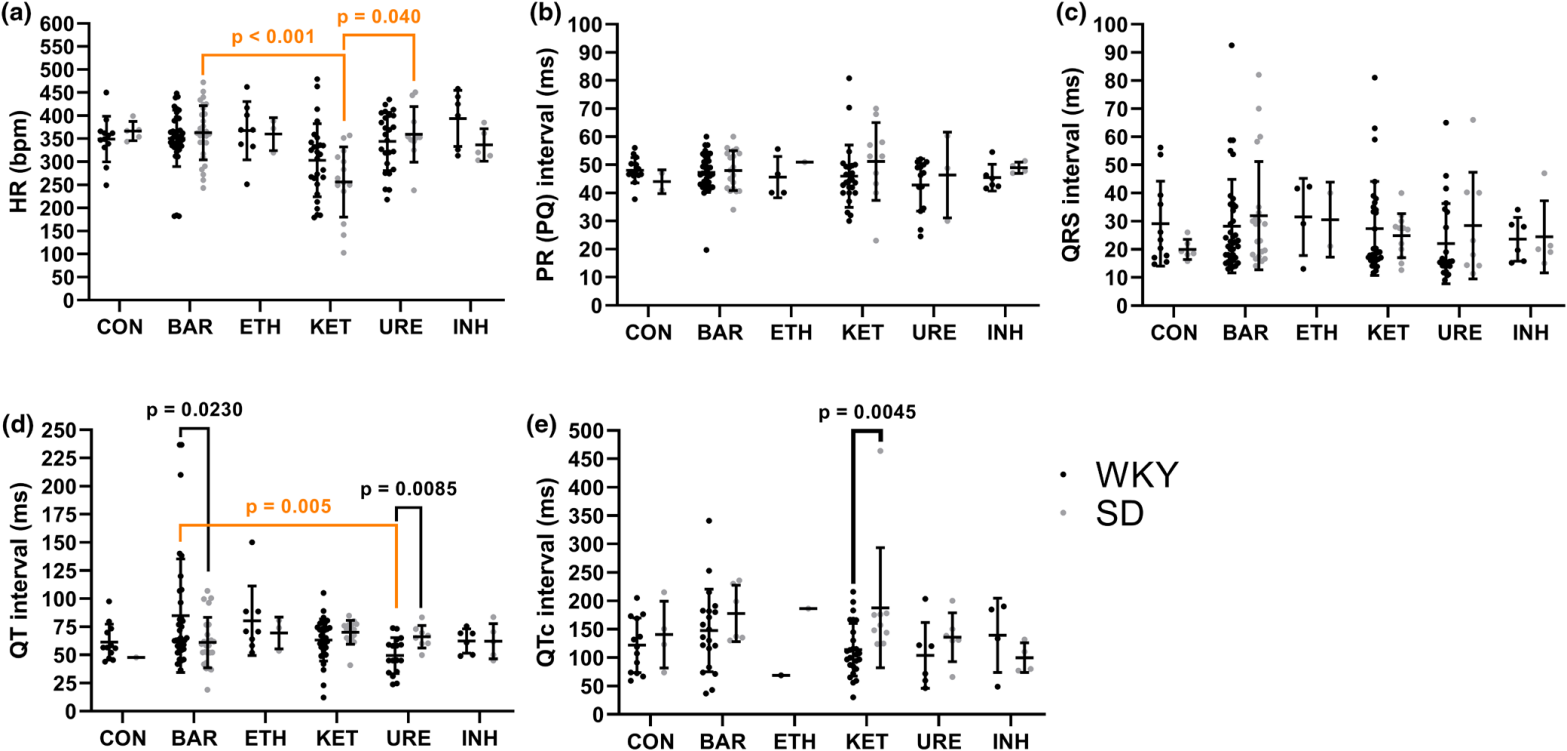
Electrocardiographic parameters in male WKY and SD rats under the most commonly used anesthetic protocols. Differences in (a) HR, (b) PR (PQ) interval, (c) QRS complex, (d) QT interval, and (e) QTc interval under various anesthetics categorized into broader groups as defined in the methodology (CON, BAR, ETH, KET, URE, and INH) are shown as data points (black points – WKY strain; gray points – SD strain) representing individual extracted mean values from available studies, with a black straight line indicating group means ± standard deviation. For clarity, the number of extracted means per group is reported in Table 6. Black bolded *p* values indicate significant strain‐dependent differences (nonparametric Mann–Whitney test), whereas orange bolded *p* values indicate significant differences between anesthetic groups within the WKY or SD strain (Dunn's post hoc test following the nonparametric Kruskal–Wallis test). BAR, barbital; bpm, beats per minute; CON, conscious rats; ETH, ether; HR, heart rate; INH, inhalation anesthesia; KET, ketamine; ms, milliseconds; SD, Sprague–Dawley; URE, urethane; WKY, Wistar or Wistar/Kyoto.

Regarding the effects of different anesthetic protocols on ECG parameters within each strain (WKY or SD male rats), the Kruskal–Wallis test revealed a significant impact of the anesthesia in the WKY strain, specifically for HR and the QT interval, and in the SD strain only for HR (Kruskal–Wallis test: *p*_WKY_(HR) = 0.014; *p*_WKY_(PR (PQ) interval) = 0.385; *p*_WKY_(QRS complex) = 0.146; *p*_WKY_(QT interval) = 0.008; *p*_WKY_(QTc interval) = 0.320; *p*_SD_(HR) < 0.001; *p*_SD_(PR (PQ) interval) = 0.852; *p*_SD_(QRS complex) = 0.684; *p*_SD_(QT interval) = 0.181; *p*_SD_(QTc interval) = 0.132). The subsequent Dunn's multiple comparison post hoc test identified, in WKY rats, a significant difference only in the QT interval between the barbital and urethane groups (with a longer QT interval under BAR; *p* = 0.005). In SD rats, a significant difference in HR was observed between the barbital and ketamine groups (with lower HR under KET; *p* < 0.001) and between the ketamine and urethane groups (with lower HR under KET; *p* = 0.040). All other comparisons were statistically nonsignificant. Due to the large amount of data generated by the post hoc tests, only statistically significant results are reported (Figure 4).

When the specific group of anesthetic agents was considered (conscious rats, barbital, ether, ketamine, urethane, and inhalation anesthesia), differences between male WKY and SD rats were observed mainly in the QT interval. Under barbital anesthesia, the QT interval was longer in WKY rats, whereas under urethane, it was significantly longer in SD rats. WKY rats exhibited a slight, nonsignificant tendency toward longer QT intervals when conscious or anesthetized with ether, nearly identical values under inhalation anesthesia, and a modest, nonsignificant shortening after ketamine administration (Table 6).

For the QTc interval, a significant difference between WKY and SD rats was observed only under ketamine, with SD rats exhibiting longer QTc values. In other anesthetic conditions—including conscious rats, barbital, ether, and urethane groups—SD rats generally exhibited a nonsignificant tendency toward longer QTc intervals. In contrast, under inhalation anesthesia, the QTc interval was slightly shorter in SD rats than that in WKY rats. The considerable variability in QTc values may reflect differences or omissions in correction methods between studies (Table 6).

No statistically significant strain differences were observed in HR, PR (PQ) interval, or QRS complex under any anesthetic condition. On average, HR was higher in WKY rats when anesthetized with ether, ketamine, or inhalation agents. In contrast, SD rats exhibited higher HR values when conscious or anesthetized with barbital or urethane. The PR (PQ) interval was generally longer in SD rats across most conditions, except in conscious animals, in which WKY rats exhibited a longer PR (PQ) interval. The QRS complex exhibited high interindividual variability: it was longer in WKY rats when conscious, under ether, or under ketamine conditions, whereas SD rats exhibited longer QRS durations under barbital, urethane, and inhalation anesthesia (Table 6).

These findings should be interpreted with caution due to the high variability of the extracted data, the lack of standardized QTc correction methods, the broad categorization of anesthetic agents, and the limited sample size in some subgroups. Consequently, not all anesthetic groups were statistically compared (e.g., QT interval in conscious rats, QTc and PR [PQ] interval under ether). Detailed statistical results and mean values are summarized in Table 6, which reports Mann–Whitney *p* values for strain‐dependent differences in each ECG parameter between anesthetic groups.

The effect of anesthesia on extracted control values of ST segment height (elevation only), P wave duration, and P, R, and T wave amplitudes in barbital, ketamine, urethane, inhalation, and ether anesthesia, and on control values obtained in awake male WKY and SD rat strains is reported in Table 7. From a descriptive review of the mean values measured under anesthesia and compared with awake rats, a tendency toward reduced ST segment elevation, longer P wave duration, and higher P and T wave amplitudes can be observed, while the R wave amplitude does not change significantly. These conclusions should be interpreted with great caution due to the lower number of extracted data.

**TABLE 7:** Anesthesia – informative values of ST segment (elevation only), P wave duration, and P, R, and T wave amplitudes in WKY male rats according to the anesthesia used.

Anesthesia	ST segment height (mV)	P wave duration (ms)	P wave amplitude (mV)	R wave amplitude (mV)	T wave amplitude (mV)
Conscious	0.14 ± 0.07 (*n* = 2)	17.98 ± 3.61 (*n* = 4)	0.07 ± 0.02 (*n* = 3)	0.61 ± 0.43 (*n* = 4)	0.09 ± 0.03 (*n* = 2)
Barbital	0.05 ± 0.05 (*n* = 17)	20.08 ± 7.27 (*n* = 12)	0.17 ± 0.09 (*n* = 12)	1.13 ± 1.32 (*n* = 17)	0.34 ± 0.50 (*n* = 14)
Ketamine	0.07 ± 0.09 (*n* = 20)	23.33 ± 7.54 (*n* = 20)	0.07 ± 0.06 (*n* = 14)	0.59 ± 0.51 (*n* = 16)	0.13 ± 0.12 (*n* = 16)
Urethane	0.08 ± 0.12 (*n* = 14)	21.17 ± 5.78 (*n* = 10)	0.077 ± 0.09 (*n* = 7)	0.49 ± 0.14 (*n* = 8)	0.19 ± 0.17 (*n* = 8)
Inhalation anesthesia	—	29.79 ± 7.83 (*n* = 6)	0.11 ± 0.07 (*n* = 4)	0.69 ± 0.88 (*n* = 3)	0.14 ± 0.04 (*n* = 3)
Ether	0.099 ± 0.12 (*n* = 2)	23.25 ± 5.30 (*n* = 2)	0.03 ± 0.0 (*n* = 1)	0.61 ± 0.46 (*n* = 2)	0.2 ± 0.0 (*n* = 1)

*Note*: Data are presented as mean ± standard deviation, along with the number of extracted studies (*n*).

In addition, other potentially relevant variables, such as anesthetic dose, depth of anesthesia, temperature, ventilation, and experimental timing, were not systematically considered, which limited our ability to determine an independent effect of anesthesia on baseline ECG parameters.

### 3.5 Sex

Another important parameter that can affect the validity of experimental studies is sex. We selected 19 studies from the databases reporting numerical descriptions of some ECG parameters: 8 for WKY (Hamed et al., 2010; Hamed et al., 2019; Lujan et al., 2007; Miranda et al., 2007; Palozi et al., 2020; Saklani et al., 2016; Saleh et al., 2020; Sosedova et al., 2019); 7 for SD (Avula et al., 2014; Coleman et al., 1987; Ketabchi et al., 2018; Peng et al., 2014; Volk et al., 2001; Wexler et al., 1973; Wexler et al., 1974); 2 for Long‐Evans female rats (Kopp et al., 1978; Kopp et al., 1980); and 1 for female Lewis rats (Yaghouby et al., 2020).

Therefore, statistical analysis could not be performed for females due to the limited data available in the studies. For the same reason, differences between sexes could not be statistically evaluated. Nevertheless, descriptive mean ECG values for both male and female WKY and SD rats, regardless of the type of anesthesia used or the QTc correction method applied, are provided only for informational purposes (Table 8). In the case of strain‐dependent differences in females, the mean HR and PR (PQ) interval values were higher in SD females, whereas the QRS complex, QT, and QTc intervals were longer in female WKY rats. When examining sex‐related differences, female WKY rats exhibited lower mean HR values than both male WKY and SD rats. In contrast, female SD rats exhibited higher HR values compared with males of both strains. For the PQ (PR) interval, female WKY rats exhibited shorter intervals than male WKY and SD rats, whereas female SD rats exhibited a shorter PQ (PR) interval than male WKY rats but longer compared with SD males. The duration of the QRS complex was shorter in both female WKY and SD rats than that in males of the corresponding strains. In contrast, the QT interval was longer in females of both normotensive strains compared with WKY and SD males. Regarding the QTc interval (regardless of the correction formula used), female WKY rats exhibited longer QTc intervals than male WKY and SD rats, whereas SD females exhibited notably shorter QTc intervals than males of both strains. These data should be interpreted with caution due to the small number of extracted datasets and other limitations, similar to those discussed for males. A graphical representation of the differences between female WKY and SD rats is presented in Figure 3.

**TABLE 8:** Comparison of mean HR values in male and female WKY and SD rats highlighting the underrepresentation of females in experimental studies, regardless of ECG recording method and anesthesia used.

Parameter	WKY	SD
HR (beats/min) – male	345.60 ± 69.66 (*n* = 125)	339.00 ± 72.15 (*n* = 70)
HR (beats/min) – female	317.50 ± 58.94 (*n* = 4)	351.70 ± 30.33 (*n* = 6)
PQ (PR) interval (ms) – male	45.94 ± 8.48 (*n* = 98)	48.65 ± 8.93 (*n* = 42)
PQ (PR) interval (ms) – female	47.5 ± 4.95 (*n* = 2)	52.40 ± 10.69 (*n* = 5)
QRS complex (ms) – male	26.63 ± 14.05 (*n* = 114)	27.32 ± 14.99 (*n* = 58)
QRS complex (ms) – female	24.45 ± 13.87 (*n* = 3)	17.81 ± 0.27 (*n* = 2)
QT interval (ms) – male	61.54 ± 21.52 (*n* = 106)	63.28 ± 15.40 (*n* = 59)
QT interval (ms) – female	68.33 ± 21.07 (*n* = 3)	66.72 ± 19.68 (*n* = 4)
QTc interval (ms) – male	125.50 ± 62.67 (*n* = 73)	158.00 ± 71.22 (*n* = 31)
QTc interval (ms) **–** female	164.90 ± 70.07 (*n* = 4)	60.00 ± 0.00 (*n* = 1)

*Note*: Descriptive electrocardiographic (ECG) data (HR, PR (PQ) interval, QRS complex, QT interval, and QTc interval, irrespective of correction method) are presented as mean ± standard deviation, along with the number of extracted studies (*n*).

Abbreviations: HR, heart rate; ms, milliseconds; *n*, number of extracted mean values from selected studies; SD, Sprague–Dawley; WKY, Wistar or Wistar/Kyoto.

Virtually, no information regarding sex differences was obtained from studies in which experiments were performed on both sexes, but without distinguishing between sexes, which represents a significant limitation to the interpretation of the results of in vivo studies (Abdel‐Hady et al., 2021; Elkholey et al., 2021; Kela et al., 1980; Krandycheva et al., 2006; Lasheen & Mohamed, 2016; Sawant et al., 2012; Shikalgar & Naikwade, 2018). However, the physiological significance of sex differences is not yet fully understood; thus, further studies are needed to clarify these mechanisms.

### 3.6 Chronobiology

The effect of light and darkness on the physiological functions of rats is one of the most important external factors. In acute in vivo experiments, the time of day at which the experiments are performed is often not considered, although the adaptation of animals to the LD cycle (12 h of light and 12 h of darkness) is usually described in the methodologies. If it is assumed that the experiments were performed during standard working hours, without acknowledgement of the influence of the LD cycle, the obtained results are then primarily from the inactive (light) period of the rat regime day. Telemetry studies that reveal circadian‐dependent changes in ECG parameters and provide reference values could facilitate the interpretation of results. However, it is highly speculative to use the values from the cited telemetry studies as reference values (even though the ECG was measured in non‐anesthetized rats).

Although there are few chronobiological studies, it has been suggested that ECG parameters probably fluctuate in a circadian dependence. Analysis of the control HR from telemetry studies involving non‐anesthetized rats, in which the influence of the LD cycle on monitored biosignals was accepted, confirmed that there is a circadian HR rhythm in rats with higher values during the active (i.e., dark) period, not only in males (Farraj et al., 2009; Hashimoto et al., 2001; Koresh et al., 2016; Mamalyga, 2014; Molcan et al., 2013; Molcan et al., 2014), but also in females (Koresh et al., 2016; Schlatter & Zbinden, 1982). A longer QTc interval was observed between 12:00 and 20:00 in WKY rats (Mamalyga, 2014). Differences were also observed in the control values of the QRS complex and PR interval, with longer durations in the afternoon than in the morning and evening hours. The QTc interval was shortened, with the lowest value observed in the evening hours (Farraj et al., 2009). It has also been shown in pentobarbital‐anesthetized female rats that cardiac electrical stability is greatest with increased HR in the dark (i.e., active) part of the regime day, when myocardial vulnerability to ventricular arrhythmias is reduced (Svorc et al., 2003).

The question is whether there are also sex differences in circadian fluctuations of ECG parameters and to what extent anesthesia can affect this. In female WKY rats, HR changes depended on the LD cycle; however, LD differences were modified by the anesthetic used. In females under ketamine/xylazine anesthesia, significant LD differences in HR and in the duration of the RR interval, PQ interval, QRS complex, QT and QTc interval were maintained (Svorc et al., 2014), in contrast to pentobarbital anesthesia, in which there were no significant LD differences (Svorc Jr et al., 2015). In female Wistar rats, pentobarbital likely only modifies circadian rhythms in ECG parameters, rather than completely disrupting them. Experiments at our institute on female rats under different types of anesthesia indicate fluctuations in ECG parameters depending on the period of the LD cycle (Table 9) (Svorc et al., 2016; Svorc & Svorc Jr, 2022).

**TABLE 9:** Mean values of ECG parameters of female Wistar rats depending on the LD cycle under selected types of anesthesia.

Parameter	Light period	Dark period	Anesthesia
HR (bpm)			
Svorc et al. (2014)	230 ± 23	276 ± 29	Ketamine/xylazine
Svorc Jr et al. (2015)	349 ± 32	390 ± 40	Pentobarbital
Svorc et al. (2016)	337 ± 16	433 ± 33	Zoletil
RR interval (ms)			
Svorc et al. (2014)	269.1 ± 37.2	213.2 ± 19	Ketamine/xylazine
Svorc Jr et al. (2015)	177 ± 15	165 ± 22	Pentobarbital
Svorc et al. (2016)	172 ± 25	219 ± 57	Zoletil
PQ interval (ms)			
Svorc et al. (2014)	47 ± 11.3	36.5 ± 5.8	Ketamine/xylazine
Svorc Jr et al. (2015)	44.16 ± 7.7	45.30 ± 4.65	Pentobarbital
Svorc et al. (2016)	51.75 ± 5.43	46.00 ± 3.44	Zoletil
QRS complex (ms)			
Svorc et al. (2014)	30 ± 2.6	26.6 ± 2.9	Ketamine/xylazine
Svorc Jr et al. (2015)	28.47 ± 2.74	28.56 ± 2.54	Pentobarbital
Svorc et al. (2016)	28.56 ± 3.54	29.17 ± 3.23	Zoletil
QT interval (ms)			
Svorc et al. (2014)	89.9 ± 16.9	91.7 ± 9.7	Ketamine/xylazine
Svorc Jr et al. (2015)	73.47 ± 15.35	76.02 ± 9.66	Pentobarbital
Svorc et al. (2016)	79.36 ± 12.27	70.66 ± 10.37	Zoletil
QTc interval (ms)			
Svorc et al. (2014)	174.5 ± 34.8	202.1 ± 20.9	Ketamine/xylazine
Svorc Jr et al. (2015)	197.72 ± 40.94	190.72 ± 26.64	Pentobarbital
Svorc et al. (2016)	200.7 ± 28	160.7 ± 33.2	Zoletil

*Note*: Electrocardiographic (ECG) data (HR, RR interval, PQ interval, QRS complex, QT interval, and QTc interval (Bazett formula)), irrespective of correction method, are presented as mean ± standard deviation.

Abbreviations: bpm, beats per minute; HR, heart rate; ms, milliseconds.

In conclusion, if HR, together with other ECG parameters, exhibits circadian fluctuations, then this fact should be accounted for in in vivo studies. However, due to inconsistent and incomplete reporting across the included studies regarding the specific period of the light–dark cycle and the exact time of day during which recordings were performed, a systematic synthesis or quantitative analysis of these chronobiological parameters was not feasible. Detailed information regarding the animals' adaptation to the LD cycle and the timing of the experiments should be provided in the methods, and the conclusions drawn should acknowledge this.

### 3.7 ST segment

The ST segment represents the time when the ventricles are depolarized and is defined as the time from the end of the QRS complex to the beginning of the T wave. The normal rat ECG profile is characterized by the absence of the ST segment. This fact is responsible for certain false patterns resembling ST segment deviations and the wide S wave in complete right bundle branch block (Sambhi & White, 1960). In control values, not only elevations of the ST segment were noted, with a mean value in WKY rats of 0.078 ± 0.09 mV (Abdolghaffari et al., 2015; Ahmed et al., 2020; Ahmed et al., 2021; Al‐Ali et al., 2024; Amin et al., 2021; Asghari et al., 2017; Aygun & Gul, 2019; Barary et al., 2022; Basol et al., 2019; Bello et al., 2023; Dos Santos et al., 2025; Doss & Kuberapandian, 2021; El‐Fawal et al., 2019; Elkattawy et al., 2023; El‐Marasy et al., 2020; El‐Wakf et al., 2024; Farouk et al., 2025; Gao et al., 2007; Hanna et al., 2022; Haydari et al., 2020; Huang et al., 2020; Jafari et al., 2015; Joukar, 2012; Karim et al., 2024; Khorrami et al., 2014; Kuberapandian & Doss, 2021; Maleki et al., 2019; Maqoud et al., 2020; Mohamed et al., 2026; Muthukumar et al., 2024; Omar et al., 2021; Parasuraman et al., 2011; Rahimi Kakavandi et al., 2022; Samadi et al., 2021; Sonawane et al., 2018; Sumbul & Aygun, 2022; Tiwari et al., 2009; Upaganlawar & Balaraman, 2010; Yadav et al., 2018; Younis et al., 2020; Younis & Mohamed, 2022; Zangiabadi et al., 2025), and with a mean value for SD rats of 0.047 ± 0.061 mV (Ali et al., 2023; Ammar el et al., 2013; Çetin, 2019; El‐Said et al., 2019; Eltobshy et al., 2023; Hu et al., 2015; Khalifa et al., 2022; Ping et al., 2021; Shahsavand et al., 2020; Shehata et al., 2025; Su et al., 2021; Wang et al., 2021; Zhang et al., 2022), but also depression (Agarwal et al., 2021; Al‐Asoom et al., 2014; Goncharov et al., 2023; Ivonin et al., 2022b; Mahmoud et al., 2024; Mostafa et al., 2024; Rahimi et al., 2018) with an average value for WKY rats of −0.059 ± 0.09 mV. Although there are probably no significant differences between the strains, they may probably be slightly lower in SD rats. No dependence on the age of the animals was observed, although there is a study that indicated that in older animals, the elevation of the ST segment is lower than in young ones (Strutynska et al., 2025). The duration of the ST segment from the selected works has a relatively large dispersion and it is problematic to perform quantitative statistical processing; therefore, the above conclusions should be regarded only as descriptive.

### 3.8 P wave

In cardiology, the P wave on ECG represents the depolarization of the atria that leads to atrial contraction, or atrial systole, and if it precedes each QRS complex in all cardiac cycles, it is sinus rhythm. However, experimental data remain lacking in available rat studies to enable definitive conclusions about sex‐, age‐, and circadian‐related changes in P wave amplitude and duration (Svorc Jr & Svorc, 2022). Regarding the comparison between rat strains, mean P wave duration (WKY, 22.49 ± 8.26 ms vs. SD, 23.08 ± 7.64 ms) and P wave amplitude (WKY, 0.099 ± 0.092 mV vs. SD, 0.113 ± 0.071 mV) showed similar values, with only slight numerical variations between WKY and SD rats.

Numerical differences between sexes were also observed after summarizing and averaging available control values from females (Abohashem et al., 2024; Hamed et al., 2010; Ketabchi et al., 2018; Miranda et al., 2007; Saleh et al., 2020; Zhao et al., 2024) and males (Aimoto et al., 2022; Akila et al., 2017; Al‐Ali et al., 2024; Ali et al., 2003; Amin et al., 2021; Arini et al., 2018; Aygun & Gul, 2019; Basol et al., 2019; Bastawy et al., 2024; Bello et al., 2023; Bestetti et al., 1987; Bilginoğlu et al., 2014; Binu et al., 2017; Botelho et al., 2019; Boushra et al., 2025; Cheng et al., 2019; Comerma‐Steffensen et al., 2017; Corydon et al., 2020; da Silva Ferreira et al., 2022; De Paula et al., 2017; Dos Santos et al., 2025; El Agaty & Seif, 2015; Farouk et al., 2025; Firoz et al., 2011; Ghelfi et al., 2008; Guéguen et al., 2008; Gupta et al., 2018; Hadova et al., 2021; Hunsaker et al., 1977; Joukar, 2012; Krandycheva et al., 2006; Lin et al., 2021; Maatz et al., 2009; Mamalyga, 2013; Maqoud et al., 2020; Martín Barrasa et al., 2008; Mohamed et al., 2026; Morand et al., 2018; Mutiso et al., 2014; Ozturk & Altug, 2007; Patel & Brocks, 2010; Ping et al., 2021; Sawant et al., 2012; Sedmera et al., 2016; Shahsavand et al., 2020; Sumbul & Aygun, 2022; Tylicki et al., 2008; Wu et al., 2023; Yadav et al., 2018; Yamanushi et al., 2014; Yang et al., 2019; Yang et al., 2020; Younis et al., 2020; Younis & Mohamed, 2022; Zangiabadi et al., 2025; Zhang et al., 2022), regardless of breed and type of anesthesia, with higher mean values in P wave duration in females (male 22.67 ± 8.02 ms vs. female 28.83 ± 12.21 ms), and with greater P wave amplitude in males (male, 0.103 ± 0.08 mV vs. female, 0.069 ± 0.01 mV). A study that directly compared sex differences in P wave duration and amplitude found higher values in female WKY rats under ketamine/xylazine anesthesia (Palozi et al., 2020). Similar to the limitations noted for strain comparisons, marked methodological heterogeneity across the included studies prevents attributing these numerical differences solely to sex.

P wave duration increases with age in both sexes (Elkholey et al., 2021; Xu et al., 2010). P‐wave amplitude decreases with age not only in ether (Mulvaney & Seronde JR., 1979) but also in pentobarbital anesthesia (Machida et al., 1990).

### 3.9 R wave

The assessment of the positive R wave in the QRS complex is also undoubtedly important because it provides information about the maximum vector of ventricular depolarization.

Sex differences in R wave amplitude have been shown in WKY rats under pentobarbital anesthesia (Wexler et al., 1973; Wexler & Greenberg, 1974) as well as in SD rats under ketamine/xylazine anesthesia (Palozi et al., 2020), with higher values in females. The available data indicate that the amplitude of the R wave decreases with age under pentobarbital (Machida et al., 1990; Wexler & Greenberg, 1974) and urethane anesthesia (Strutynska et al., 2025). In ether anesthesia, it increases with age; however, in 11–12 week‐old animals, it decreases (Mulvaney & Seronde JR., 1979).

In male WKY rats, the mean R‐wave amplitude was shorter than that in SD rats (WKY, 0.63 ± 0.43 mV vs. SD, 1.072 ± 1.44 mV).

### 3.10 T wave

The T wave is the most variable wave on the ECG profile and represents ventricular repolarization.

Potential sex differences in T wave amplitude have previously been suggested, with higher amplitudes reported in females (Palozi et al., 2020; Wexler et al., 1973). A similar pattern was observed in our averaged data (males, 0.204 ± 0.29 mV vs. females, 0.22 ± 0.05 mV). Regarding strain variations, mean T wave amplitude also appeared numerically higher in SD rats compared to WKY rats (WKY, 0.16 ± 0.12 mV vs. SD, 0.29 ± 0.49 mV), aligned with the trends seen for P and R waves. However, as with the previous parameters, these numerical differences serve strictly descriptive purposes due to inter‐study methodological heterogeneity.

### 3.11 Study limitations

Regarding why some results are statistically significant, and others are not, despite apparent differences in mean values, several possible explanations need to be considered. These may include the very high variability of the data (in the case of QTc, influenced by, for example, inconsistencies in correction methods), non‐independence, the lack of weighting, the varying degree of stress placed on the rats during measurement, aggregation of study‐level mean values into a single mean, the inclusion of different effective anesthetics into one broad category, combination of Wistar‐Kyoto and Wistar rats into one strain group, in some cases very small datasets (*n* = 1) or no data, or significantly unequal sample sizes between some of the groups compared. The use of non‐parametric tests that assess differences in the distribution of values (or medians) and not directly in the means may also have contributed to the nonsignificant results. As such, apparent differences in the mean values may not yield statistical significance in these tests. Nonparametric tests were used in our case due to the nonnormal distribution of the data, small and uneven number of data and high variability, which could violate the assumptions of parametric analysis of variance. In addition, our grouping of different effective anesthetics into broader categories may neglect some specific effects of individual agents, which we acknowledge as a limitation of our analysis.

## 4 CONCLUSIONS

Baseline or control ECG parameters in rats are frequently used as reference points in experimental research, yet they are susceptible to substantial biological and methodological variability prior to any intervention. This systematic review summarized baseline or control ECG parameters to identify potential biological and methodological sources of variability or confounding affecting them. Due to widespread methodological heterogeneity, incomplete reporting in the primary literature, and necessary analytical limitations, including study‐level data aggregation and confounding, firm conclusions regarding specific physiological determinants (e.g., age, weight, or strain differences) cannot be definitively established. Moreover, the lack of standardized reporting for light–dark cycles and the routine application of unvalidated QTc correction formulas further complicate data interpretation. Therefore, standardized reporting practices and further systematic investigations are essential to establish reliable reference frameworks for rat electrocardiography.

## AUTHOR CONTRIBUTIONS

**Viktória Novotná:** Data curation; formal analysis; investigation; methodology; software; validation; visualization. **Soňa Grešová:** Investigation; validation. **Pavol Švorc:** Conceptualization; data curation; formal analysis; investigation; methodology; software; supervision; validation.

## FUNDING INFORMATION

No funding information provided.

## CONFLICT OF INTEREST STATEMENT

No conflicts of interest.

## ETHICS STATEMENT

This systematic review does not include any animal experiments conducted by the authors. Therefore, ethical approval was not required.

## REVIEW REGISTRATION

The authors declare that this systematic review was not registered in a prospective registry before its initiation. Therefore, no preexisting protocol is available.

## Supporting information


Data S1.

## Data Availability

All summarized and analyzed data are included in this study. Extracted mean values from individual studies are available from the corresponding author upon reasonable request.

## Footnotes

^1^
MSD Veterinary Manual. Resting heart rates [table]. Available from: https://www.msdvetmanual.com/multimedia/table/resting‐heart‐rates.
